# A Novel Approach to Gastrointestinal Bleeding Risk Stratification and Proton Pump Inhibitor Effectiveness in Patients with Acute Coronary Syndrome on Dual Antiplatelet Therapy: A Nationwide Retrospective Cohort Study

**DOI:** 10.1007/s10557-025-07702-4

**Published:** 2025-04-26

**Authors:** Mee Yeon Lee, Kyu-Nam Heo, Jaekyu Shin, Ju-Yeun Lee

**Affiliations:** 1 College of Pharmacy and Research Institute of Pharmaceutical Sciences, Seoul National University, 1, Gwanak-Ro, Gwanak-Gu, Seoul, 08826 Republic of Korea; 2 Department of Clinical Pharmacy, School of Pharmacy, University of California, San Francisco, CA 94607 USA

**Keywords:** Bleeding risk stratification, Dual antiplatelet therapy (DAPT), Gastrointestinal bleeding, Modified Academic Research Consortium High Bleeding Risk (mARC-HBR) criteria, Proton pump inhibitors (PPI)

## Abstract

**Purpose:**

Proton pump inhibitors (PPIs) are effective in preventing gastrointestinal (GI) bleeding in high-risk patients on dual antiplatelet therapy (DAPT). Existing criteria for high GI bleeding risk, such as those from the American Heart Association (AHA), may not fully reflect East Asian patient profiles. This study aimed to evaluate the effectiveness of PPIs in preventing GI bleeding across DAPT combinations, stratified by GI bleeding risk using the Academic Research Consortium High Bleeding Risk (ARC-HBR) criteria in patients with acute coronary syndrome (ACS).

**Methods:**

A retrospective cohort of 93,153 patients with ACS initiating DAPT (2018–2020) was analyzed using the Korean National Health Insurance database. Modified ARC-HBR (mARC-HBR) criteria tailored to claims data were compared with AHA criteria in terms of concordance and performance. PPI effects on GI bleeding were analyzed by mARC-HBR risk groups over a 3-year observation period.

**Results:**

The mARC-HBR criteria identified three times more high-risk patients than the AHA criteria, demonstrating higher sensitivity (38.9% vs. 11.1%, *p* < 0.001) while maintaining a relatively high specificity (both > 70%). While PPI use offered no benefit for low-risk patients, it was associated with a 25.8% lower GI bleeding risk in high-risk patients, with the most pronounced effect observed in those on the aspirin/ticagrelor combination.

**Conclusion:**

The mARC-HBR criteria enhance the identification of high GI bleeding risk patients with ACS and may inform targeted PPI use, given the observed associations suggesting potential benefit in high-risk ticagrelor users and limited effect in low-risk groups.

**Supplementary Information:**

The online version contains supplementary material available at 10.1007/s10557-025-07702-4.

## Introduction

Dual antiplatelet therapy (DAPT) is crucial for acute coronary syndrome (ACS) management [1] but increases gastrointestinal (GI) bleeding risk, with incidence rates reaching 3.9% within 30 days [2]. Aspirin/clopidogrel (AC) raises bleeding risk 2.61-fold compared to aspirin alone [3], while the TRITON-TIMI 38 trial and meta-analyses show higher risks with aspirin/prasugrel (AP) (hazard ratio [HR] 1.32, *p* = 0.03) and aspirin/ticagrelor (AT) (relative risk [RR] 1.40, *p* = 0.01) compared to AC, respectively [4, 5]. GI bleeding during DAPT is linked to 4.87-fold higher 30-day mortality, underscoring the need for preventive strategies [6].

Proton pump inhibitors (PPIs) significantly reduce GI bleeding risk during DAPT (RR 0.37–0.51) [7] and improve survival with a 67% reduction in all-cause mortality [8] but raise concerns about potential drug interactions with clopidogrel that may reduce antiplatelet efficacy [9], as well as long-term adverse effects such as acute kidney injury, hypomagnesemia, and gastric cancer [10]. Current guidelines for PPI use during DAPT vary, with American Heart Association (AHA) guidelines recommending use in high-risk patients [11] and European Society of Cardiology (ESC) guidelines advocating routine use for all [12].

This lack of unified guidance results in inconsistent practices, with only 47.6% of the US patients receiving appropriate PPI prescriptions—29.6% underuse in high-risk and 22.8% overuse in low-risk groups [13]. In South Korea, PPIs are prescribed to only half of high-risk myocardial infarction (MI) patients, while 35.8% of low-risk patients receive them unnecessarily [14]. These findings reveal a gap between guidelines and real-world practice, emphasizing the need for tailored approaches that strengthen PPI use in high-risk patients while minimizing overuse in low-risk groups.

However, these recommendations for PPI use during DAPT are based on Western populations, raising concerns about their applicability to Asians, who face higher bleeding risks and lower ischemic event rates [15]. Asians had a 2.26-fold higher bleeding risk (HR 2.26, 95% confidence interval [CI] 1.22–4.20) in randomized trials, with major bleeding incidence at 0.6% compared to 0.3% in Westerners [16]. Studies in Chinese and Korean populations revealed inconsistencies between AHA-guideline risk stratification and actual bleeding rates, indicating underestimated risks in Asians [14, 17]. Genetic and pharmacokinetic differences, such as reduced CYP450 activity, exacerbate bleeding risks in East Asians, affecting clopidogrel metabolism and contributing to the “East Asian paradox”—a phenomenon which shows lower ischemic event risks with disproportionately higher bleeding risks [18]. Stronger P2Y12 inhibitors like prasugrel and ticagrelor further elevate bleeding risks due to 40–50% higher active metabolite levels in Asians [19]. These disparities underscore the limitations of Western-derived guidelines, highlighting the need for tailored approaches, such as the Academic Research Consortium High Bleeding Risk (ARC-HBR) criteria, to better address Asian-specific bleeding profiles [14].

The ARC-HBR criteria have been validated as an effective tool for stratifying bleeding risks in Asian populations [20, 21], including those in Korea and Japan [22, 23]. Furthermore, although designed for overall bleeding risk prediction, they have approximately 70% of their risk factors clinically associated with GI bleeding [24–29], while the remaining factors may indirectly increase this risk. However, these criteria have not yet been applied to evaluate the clinical benefits of PPIs or to identify high-risk GI bleeding patients during DAPT. This lack of research highlights a critical gap, as the ARC-HBR criteria could provide a more accurate assessment of GI bleeding risks and guide optimal PPI use strategies for Asian patients.

Research on PPI effectiveness in preventing GI bleeding during DAPT is limited, especially for low-risk patients, with only two known investigations showing conflicting results: one study in Chinese patients reported an increased risk of GI bleeding with PPI use (adjusted odds ratio 5.58, 95% CI 2.90–10.70) [17], while another in Korean patients demonstrated a 46% reduction in severe GI bleeding requiring transfusion (HR 0.54, 95% CI 0.43–0.68) [14]. Additionally, most existing research focuses on AC combinations, like the COGENT trial (GI events reduced from 2.9% to 1.1%, *p* < 0.001) [30], but data on higher-risk AP and AT combinations remain scarce, despite their higher associated bleeding risks.

This study aimed to evaluate the clinical effectiveness of PPIs in preventing GI bleeding among patients with ACS receiving DAPT, using a novel approach to stratify bleeding risk with the modified Academic Research Consortium High Bleeding Risk (mARC-HBR) criteria. Furthermore, the study analyzed the effectiveness of PPIs in reducing GI bleeding across different DAPT regimens.

## Methods

### Data Source

This study utilized data from the customized National Health Insurance claims database provided by the Korean Health Insurance Review and Assessment Service (HIRA). HIRA covers 98% of the Korean population under the National Health Insurance System, providing comprehensive patient profiles including de-identified data on demographics, healthcare utilization, treatments, diagnoses, and prescriptions [31].

### Study Design and Population

This retrospective cohort study included patients hospitalized for the first time due to ACS between January 1, 2018, and June 30, 2020, who received DAPT for at least 7 days. DAPT was defined as the combination of aspirin and a P2Y12 inhibitor (clopidogrel, prasugrel, or ticagrelor), regardless of aspirin dose. Patients on warfarin or direct oral anticoagulants (DOACs) before or during hospitalization were excluded due to their associated high bleeding and MACE risk and their indicated comorbidities [32]. Those with truncated claims data within 1 year or GI bleeding during hospitalization were also excluded due to data limitations [31].

Patients were categorized into PPI users and gastroprotective agent (GPA) non-users. The PPI user was defined as patients who co-administered a GPA with DAPT and maintained continuous PPI use for at least 24 of the first 30 days (≥ 80% adherence). The GPA non-user cohort consisted of patients who did not use any GPAs (PPIs, histamine- 2 receptor antagonists [H2RAs], potassium-competitive acid blockers [P-CABs], rebamipide, *Artemisia* herb soft extract, or misoprostol) for ≥ 24 days during the first 30 days. Patients who used other GPAs (H2RAs, P-CABs, rebamipide, *Artemisia* herb soft extract, or misoprostol) exclusively for ≥ 80% of the observation period, or who cumulatively used multiple GPAs (PPIs, H2RAs, P-CABs, rebamipide, *Artemisia* herb soft extract, or misoprostol) for ≥ 80% of the observation period, were excluded.

The treatment episode initiated on the first date of the combined DAPT and GPA therapy, and ended when DAPT treatment was terminated. A gap of less than 30 days between supplies was considered as maintaining continuous use for both DAPT and GPA, respectively. For determining the end of treatment, a grace period was allowed, considering 80% adherence following the last medication supply.

We modified the internationally validated ARC-HBR criteria [20] to create the modified ARC-HBR (mARC-HBR) criteria to fit claims data constraints. Detailed operational definitions of the mARC-HBR criteria and the timeframes for assessing bleeding risk are presented in Online Resource Table 1. Major risk factors included a glomerular filtration rate (GFR) of < 30 mL/min, bleeding requiring hospitalization or transfusion within 6 months, cirrhosis with portal hypertension, active malignancy within 12 months, a history of spontaneous or traumatic intracranial hemorrhage, moderate to severe ischemic stroke within 6 months, and major surgery or trauma within 30 days. Minor factors included age ≥ 75 years, bleeding requiring hospitalization or transfusion within 12 months not meeting the major criteria, and chronic nonsteroidal anti-inflammatory drug (NSAID) or steroid use. Due to the retrospective nature of the claims database, criteria requiring specific clinical or laboratory data were excluded (Online Resource Table 1). Patients with at least one major or two minor criteria were classified as high risk, while others were classified as low risk [20]. Patients expected to require long-term oral anticoagulation therapy were excluded from the study design. Propensity score matching was applied within each risk group to compare outcomes between PPI users and GPA non-users. Key matching variables included demographic and clinical factors associated with GI bleeding risk and GPA utilization.

Baseline comorbidities, percutaneous coronary intervention (PCI) placement during hospitalization of ACS, and co-medications were adjusted in the evaluation. Comorbidities included alcoholism, chronic kidney disease (CKD), diabetes mellitus (DM), dyspepsia, gastroesophageal reflux disease, heart failure, hemorrhagic stroke, hypertension, irritable bowel disease, ischemic stroke, non-severe and severe peptic ulcer disease, thrombocytopenia, and transient ischemic attack. Comorbidities were documented based on any occurrence prior to the index date. Co-medications with the potential to elevate GI bleeding risk, including non-selective NSAIDs, cyclooxygenase-2 inhibitors, corticosteroids, and selective serotonin reuptake inhibitors were adjusted if used for more than seven cumulative days within the first 30 days following cohort entry [33–35]. To minimize potential bias from missing data, we assumed that the lack of diagnostic or prescription records represented the absence of respective comorbidities or medications.

This study was conducted and reported in accordance with the Strengthening the Reporting of Observational Studies in Epidemiology (STROBE) guidelines [36], as detailed in Online Resource Table 2.

### Outcome Definition

In this study, the primary outcome was defined as GI bleeding, identified as upper or lower GI bleeding requiring hospitalization, emergency room visits, or outpatient visits within 30 days after the treatment episode, with the earliest occurrence date of these events recorded as the outcome date. The secondary outcome was MACE, defined as a composite endpoint including cardiovascular death, acute MI, and stroke, with the outcome determined by the earliest occurrence date among these three events (Online Resource Table 3) [37].

Stroke was defined as a new diagnosis requiring emergency room visits or hospitalization, with no prior stroke diagnosis in the preceding year to avoid misclassification. Acute MI was defined as a new hospitalization with MI primary diagnosis accompanied by thrombolytic therapy (alteplase, tenecteplase, reteplase), or specific thrombolysis procedures, or underwent a PCI. Death was operationally defined as the last claim indicating death as the discharge status or no claims for over 150 days despite follow-up capability [38], and cardiovascular death was identified when cardiovascular diagnoses were listed as the primary diagnosis within 1 month prior to death [39].

Patients were followed up for a minimum of 1 year and up to 3 years after cohort entry. Censoring occurred immediately upon the earliest of the following events: 30 days after the discontinuation of continuous DAPT, the end date of the claims data, or the use of oral or intravenous anticoagulants for more than 7 days following the index hospitalization. Competing risk analysis was performed to account for mortality as a competing event.

### Comparative Analysis of mARC-HBR and AHA Criteria

To evaluate the applicability of the ARC-HBR criteria in classifying GI bleeding risk, the mARC-HBR and AHA criteria were compared using claims data. The AHA criteria define high-risk patients as those with prior GI bleeding or at least two risk factors, including advanced age (≥ 60 years), use of anticoagulants, steroids, or NSAIDs, and *Helicobacter pylori* infection [40]. Given recent findings indicating a higher GI bleeding incidence in patients aged ≥ 75 years [41], we applied the 75-year age threshold as a criterion for advanced age under the AHA criteria. Due to data constraints, anticoagulant use and *Helicobacter pylori* infection were excluded. Thus, high-risk patients under the AHA criteria were defined as those with a GI bleeding history or aged ≥ 75 years using steroids or NSAIDs. Concordance and patient characteristics under the mARC-HBR and AHA criteria were analyzed. Sensitivity, specificity, positive predictive value (PPV), and negative predictive value (NPV) were calculated, and statistical differences were assessed. A confusion matrix heatmap was generated to visually compare classification results with actual GI bleeding outcomes.

### DAPT Subgroup Analysis

A subgroup analysis assessed PPI effects on GI bleeding prevention and MACE across DAPT combinations (AC, AP, AT) using matched samples from the main analysis. Patients with multiple DAPT regimens during hospitalization were excluded due to the inability to track exact regimen switches in claims data. An intent-to-treat approach was applied, maintaining patients in their initially assigned DAPT cohort throughout follow-up, regardless of regimen changes until the end of continuous therapy. GI bleeding and MACE outcomes were compared between PPI users and GPA non-users within each risk group and DAPT regimen. In the AC group, PPI users were further classified into omeprazole/esomeprazole and other PPI users to evaluate potential cardiovascular risks associated with clopidogrel interactions.

### Statistical Analysis

Categorical variables were reported as absolute and relative frequencies. Chi-square or Fisher’s exact tests compared unmatched cohorts, and standardized mean differences (SMDs) assessed balance post-matching. Paired proportion *z*-tests compared GI bleeding risk stratification between criteria. Incidence rates for GI bleeding and MACE were calculated per total person-time at risk. Competing risk-adjusted cumulative incidence function graphs were generated using cause-specific hazard estimates for event-specific analysis and Gray’s test for cumulative incidence function comparison [42]. Propensity score matching used a greedy algorithm (caliper: 0.2 standard deviations of logit) without replacement. Adjusted HRs were estimated via cause-specific Cox proportional hazards models using GPA non-users as the reference. Subgroup analyses restricted adjustment variables to maintain statistical power given the reduced sample sizes. All statistical analyses were conducted using SAS version 6.1 (SAS Institute, Cary, NC). As a sensitivity analysis, we applied a multivariable Cox model to a pooled cohort of high- and low-risk patients, including an interaction term between PPI use and bleeding risk status. Given the heterogeneity in bleeding risk, hazard ratios were estimated within each stratum to maintain clinical interpretability [43].

## Results

### Demographic and Clinical Characteristics

**Fig. 1:**
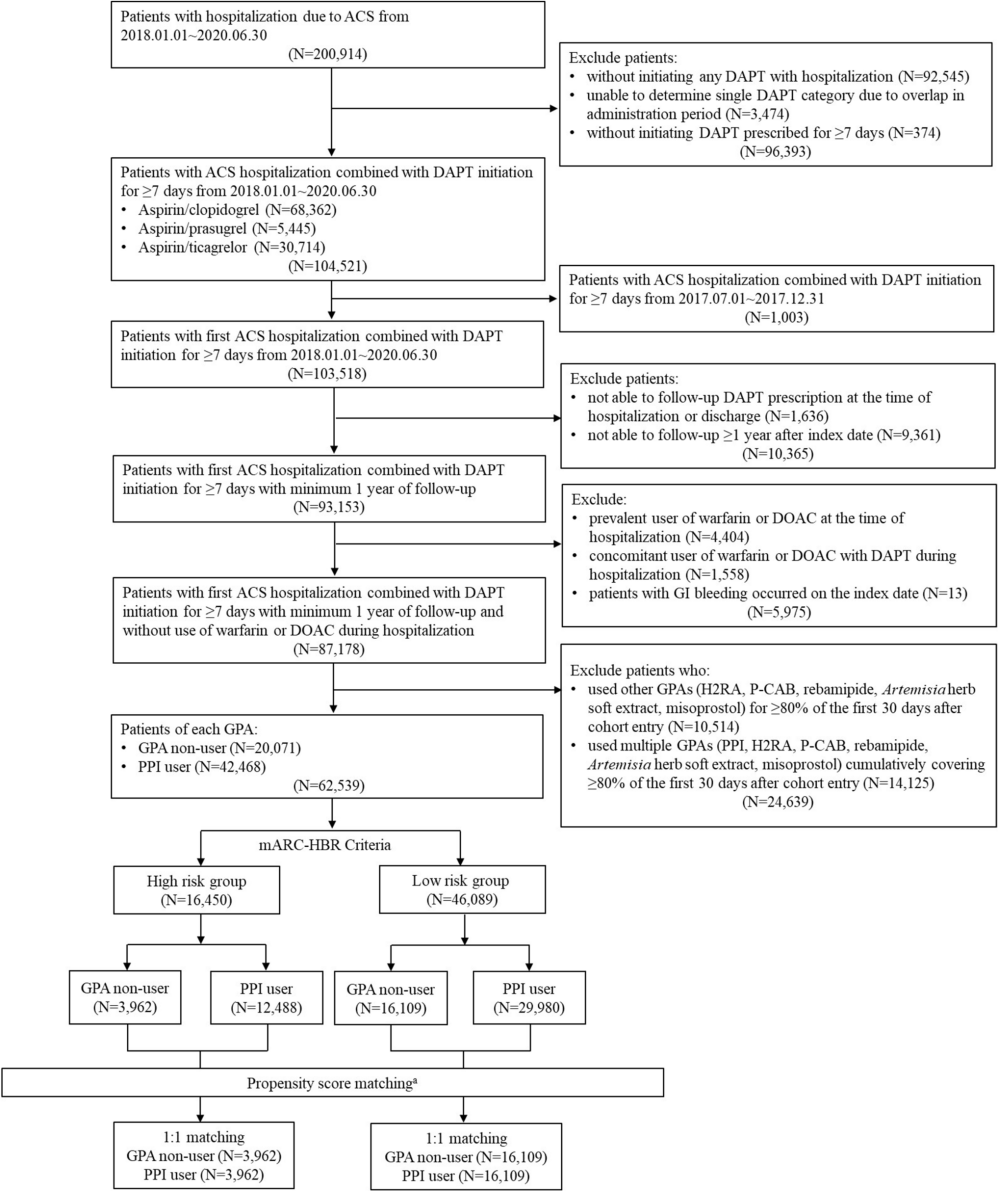
Flowchart of patient selection process. ^a^Matching variables: Charlson Comorbidity Index (CCI) group, sex, history of spontaneous bleeding requiring hospitalization or transfusion within the past 6 months, chronic non-steroidal anti-inflammatory drug or steroid use, non-severe peptic ulcer disease, ischemic stroke, and hypertension. *ACS* acute coronary syndrome; *DAPT* dual antiplatelet therapy; *DOAC* direct oral anticoagulant; *GI* gastrointestinal; *GPA* gastroprotective agents; *H2RA* histamine- 2 receptor antagonist; *P-CAB* potassium-competitive acid blocker; *PPI* proton pump inhibitor

Among an initial pool of 200,914 patients, 93,153 individuals were hospitalized for the first time due to ACS and initiated DAPT for at least 7 days between January 1, 2018, and June 30, 2020. After applying the exclusion criteria, cohorts of GPA non-users (*N* = 20,071) and PPI users (*N* = 42,468) were established. Following risk group stratification by the mARC-HBR criteria, the final matched cohorts for each stratum are shown in Fig. 1.

The demographic and clinical characteristics of each cohort and bleeding risk classification factors before propensity score matching are summarized in Online Resource Table 4 and Online Resource Table 5. AC was the most frequently prescribed DAPT regimen, and more than half of the patients underwent PCI during initial hospitalization. The PPI users had higher rates of NSAID and corticosteroid co-administration and higher Charlson Comorbidity Index scores than the GPA non-users. In the high bleeding risk group, spontaneous bleeding within 6 months and moderate to severe ischemic stroke were more common in PPI users, while GFR < 30 mL/min and active malignancy within 12 months were more frequent in GPA non-users. Across all groups, PPI users were more likely to be ≥ 75 years old and chronically use NSAIDs or corticosteroids compared to GPA non-users.

Following propensity score matching, the cohort characteristics and risk factor profiles are presented in Table 1 and Online Resource Table 6. Most variables were balanced, with SMDs below 0.1, and any residual imbalances were adjusted in multivariable models. All SMDs were below 0.3, meeting Cohen’s acceptable range for statistical validity [44].

**Table 1:** Baseline characteristics of matched cohorts of dual antiplatelet therapy users by risk grade and gastroprotective agent use (after propensity score matching)

Characteristics	High risk group (*N* = 7924)			Low risk group (*N* = 32,218)		
	GPA non-user (*N *= 3962)	PPI user (*N* = 3962)	SMD	GPA non-user (*N* = 16,109)	PPI user (*N* = 16,109)	SMD
	*N* (%)	*N* (%)		*N* (%)	*N* (%)	
Age, years						
< 65	1521 (38.4)	1367 (34.5)	0.093	10,010 (62.1)	9418 (58.5)	0.094
65–74	1089 (27.5)	1155 (29.2)		3870 (24.0)	3949 (24.5)	
75–84	1148 (29.0)	1181 (29.8)		1887 (11.7)	2326 (14.4)	
≥ 85	204 (5.2)	259 (6.6)		342 (2.1)	416 (2.6)	
Sex, male	1091 (27.5)	1091 (27.5)	0.000	13,178 (81.8)	13,178 (81.8)	0.000
Dual antiplatelet therapy types						
Aspirin/clopidogrel	2833 (71.5)	2857 (72.1)	0.052	8769 (54.4)	8888 (55.5)	0.025
Aspirin/prasugrel	151 (3.8)	114 (2.9)		1197 (7.4)	1098 (6.8)	
Aspirin/ticagrelor	978 (24.7)	991 (25.0)		6143 (38.1)	6123 (38.0)	
CCI score (mean ± SD)	3.9 ± 2.2	3.9 ± 2.3	0.000	2.0 ± 1.5	2.0 ± 1.5	0.000
0–1	544 (13.7)	544 (13.7)	0.000	6615 (41.1)	6615 (41.1)	0.000
2–4	2010 (50.7)	2010 (50.7)		8595 (53.4)	8595 (53.4)	
≥ 5	1408 (35.5)	1408 (35.5)		899 (5.6)	899 (5.6)	
PCI insertion during hospitalization	2545 (64.2)	2428 (61.3)	0.061	10,661 (66.2)	10,566 (65.6)	0.012
Comorbidities						
Alcoholism	76 (1.9)	91 (2.3)	0.026	210 (1.3)	313 (1.9)	0.051
Chronic kidney disease	958 (24.2)	806 (20.3)	0.092	84 (0.5)	88 (0.6)	0.003
Diabetes mellitus	1712 (43.2)	1621 (40.9)	0.047	4401 (27.3)	4246 (26.4)	0.022
Dyspepsia	885 (22.3)	1028 (26.0)	0.084	3110 (19.3)	3720 (23.1)	0.093
Gastroesophageal reflux disease	1313 (33.1)	1785 (45.1)	0.246	4578 (28.4)	6518 (40.5)	0.256
Heart failure	418 (10.6)	394 (9.9)	0.020	809 (5.0)	814 (5.1)	0.001
Hemorrhagic stroke	61 (1.5)	59 (1.5)	0.004	18 (0.1)	25 (0.2)	0.012
Hypertension	2519 (63.6)	2519 (63.6)	0.000	8246 (51.2)	8245 (51.2)	0.000
Irritable bowel disease	14 (0.4)	11 (0.3)	0.014	29 (0.2)	35 (0.2)	0.008
Ischemic stroke	483 (12.2)	483 (12.2)	0.000	462 (2.9)	462 (2.9)	0.000
Non-severe peptic ulcer disease	625 (15.8)	625 (15.8)	0.000	2088 (13.0)	2088 (13.0)	0.000
Severe peptic ulcer disease	45 (1.1)	84 (2.1)	0.078	74 (0.5)	109 (0.7)	0.029
Thrombocytopenia	13 (0.3)	13 (0.3)	0.000	17 (0.1)	22 (0.1)	0.009
Transient ischemic attack	103 (2.6)	117 (3.0)	0.022	225 (1.4)	261 (1.6)	0.018
Co-medications						
Corticosteroid	353 (8.9)	622 (15.7)	0.208	1096 (6.8)	1725 (10.7)	0.138
COX2 inhibitor	133 (3.4)	166 (4.2)	0.044	115 (0.7)	207 (1.3)	0.057
Ketolorac	35 (0.9)	79 (2.0)	0.093	184 (1.1)	210 (1.3)	0.015
Non-ketolorac traditional NSAID	346 (8.7)	608 (15.4)	0.204	913 (5.7)	1618 (10.0)	0.163
Selective serotonin reuptake inhibitor	144 (3.6)	182 (4.6)	0.048	250 (1.6)	359 (2.2)	0.050

Values are expressed as mean ± SD, or percentages

SMD values are presented as absolute values (|SMD|)

*CCI* Charlson Comorbidity Index, *COX2* cyclooxygenase- 2, *GPA* gastroprotective agents, *NSAID* non-steroidal anti-inflammatory drugs, *PCI* percutaneous coronary intervention, *PPI *proton pump inhibitor, *SD* standard deviation, *SMD* standardized mean difference

### Comparison Between mARC-HBR and AHA Criteria

Using mARC-HBR criteria, 16,450 patients (26.3%) were classified as high-risk and 46,089 patients (73.7%) as low-risk. In contrast, under the AHA criteria, 5652 patients (9.0%) were classified as high-risk and 56,887 patients (91.0%) as low-risk. Concordance analysis showed that 20.0% of the patients classified as high-risk by mARC-HBR criteria were also classified as high-risk by the AHA criteria, while 94.9% of the patients classified as low-risk by mARC-HBR criteria were similarly categorized by AHA. This difference reflects the simpler high-risk criteria of the AHA, with 58.2% of AHA high-risk patients also classified as high-risk by mARC-HBR.

Further analysis of discordant groups revealed that in the 2362 patients classified as high-risk by AHA criteria but low-risk by mARC-HBR criteria, all cases (100%) were attributed to GI bleeding that occurred more than 12 months before the index date. Conversely, among the 13,160 patients classified as high-risk by mARC-HBR criteria but low-risk by AHA standards, the most common factors included major surgery or trauma within the past 30 days (35.8%), spontaneous bleeding requiring hospitalization or transfusion within the past 6 months or recurrent bleeding (27.7%), and an estimated GFR < 30 mL/min (23.6%).

To compare the performance of the two criteria, the actual GI bleeding incidence rates within each risk group were analyzed. The incidence was 3.0% for patients classified as high-risk by both mARC-HBR and AHA criteria, 2.8% for those classified as high-risk by mARC-HBR but low-risk by AHA, 1.4% for those classified as high-risk by AHA but low-risk by mARC-HBR, and 1.6% for patients classified as low-risk by both criteria.

**Fig. 2:**
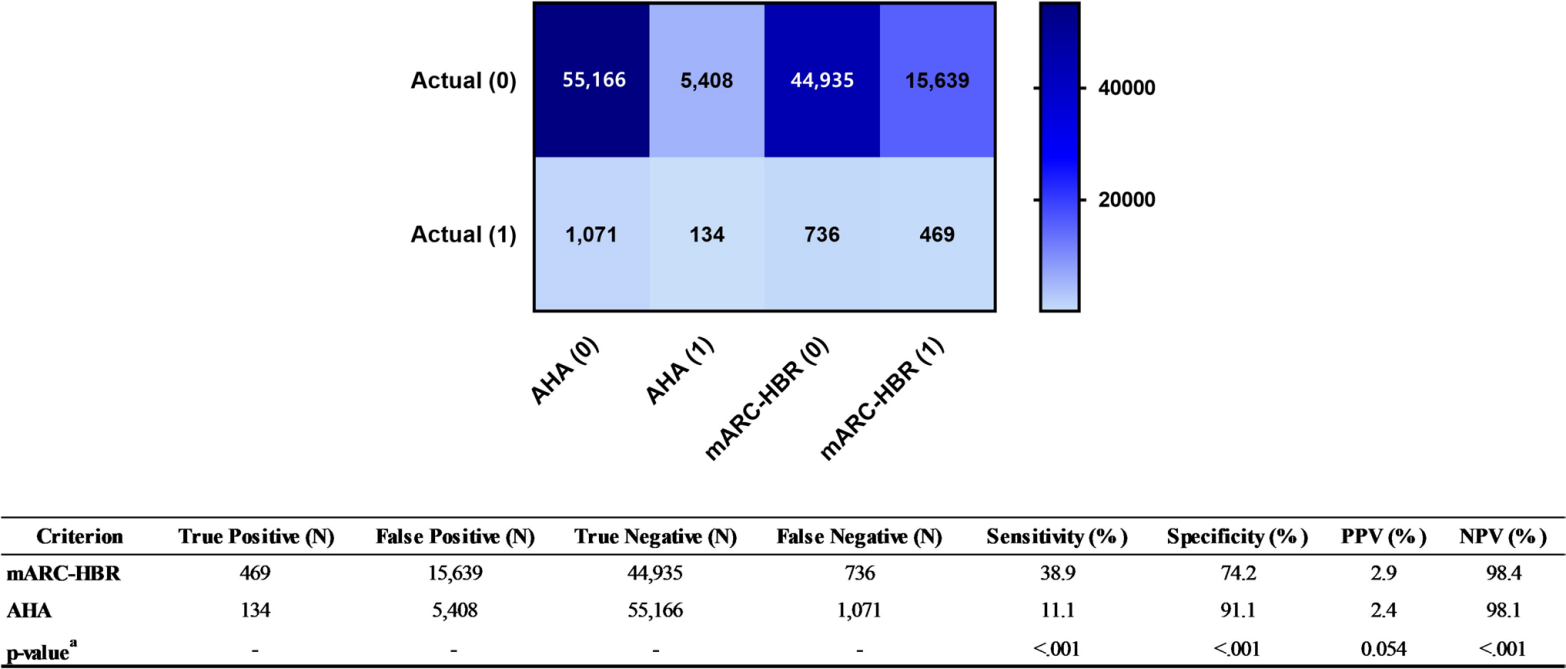
Comparison of gastrointestinal bleeding risk stratification performance between mARC-HBR and AHA criteria. The upper panel displays a confusion matrix heatmap comparing the performance of the mARC-HBR and AHA criteria for gastrointestinal bleeding risk stratification. Actual outcomes are labeled as (0) for patients who did not experience GI bleeding and (1) for those who did. The color intensity represents the count of true positives (TP), false positives (FP), true negatives (TN), and false negatives (FN) for each criterion. The lower panel provides a quantitative comparison of sensitivity, specificity, positive predictive value (PPV), and negative predictive value (NPV) for both criteria. *p*-values for these metrics were calculated using paired proportions *z*-tests. Raw counts (TP, FP, TN, FN) are presented as descriptive statistics without *p*-values. *AHA* American Heart Association; *mARC-HBR* modified Academic Research Consortium for High Bleeding Risk; *NPV* negative predictive value; *PPV* positive predictive value

Statistical comparisons and visualizations of the performance differences between the two criteria are presented in Fig. 2. The mARC-HBR criteria exhibited significantly higher sensitivity compared to the AHA criteria (38.9% vs. 11.1%, *p* < 0.001), with specificity remaining relatively high for both, exceeding 70%. The mARC-HBR criteria identified approximately 3.5 times more true-positive patients than the AHA criteria and demonstrated a lower false-negative rate, indicating an improved ability to classify high-risk patients, albeit with a higher false-positive rate. Both criteria showed low PPVs and high NPVs.

### Impact of PPI Use on GI Bleeding and MACE Outcomes in High-Risk Patients

**Fig. 3:**
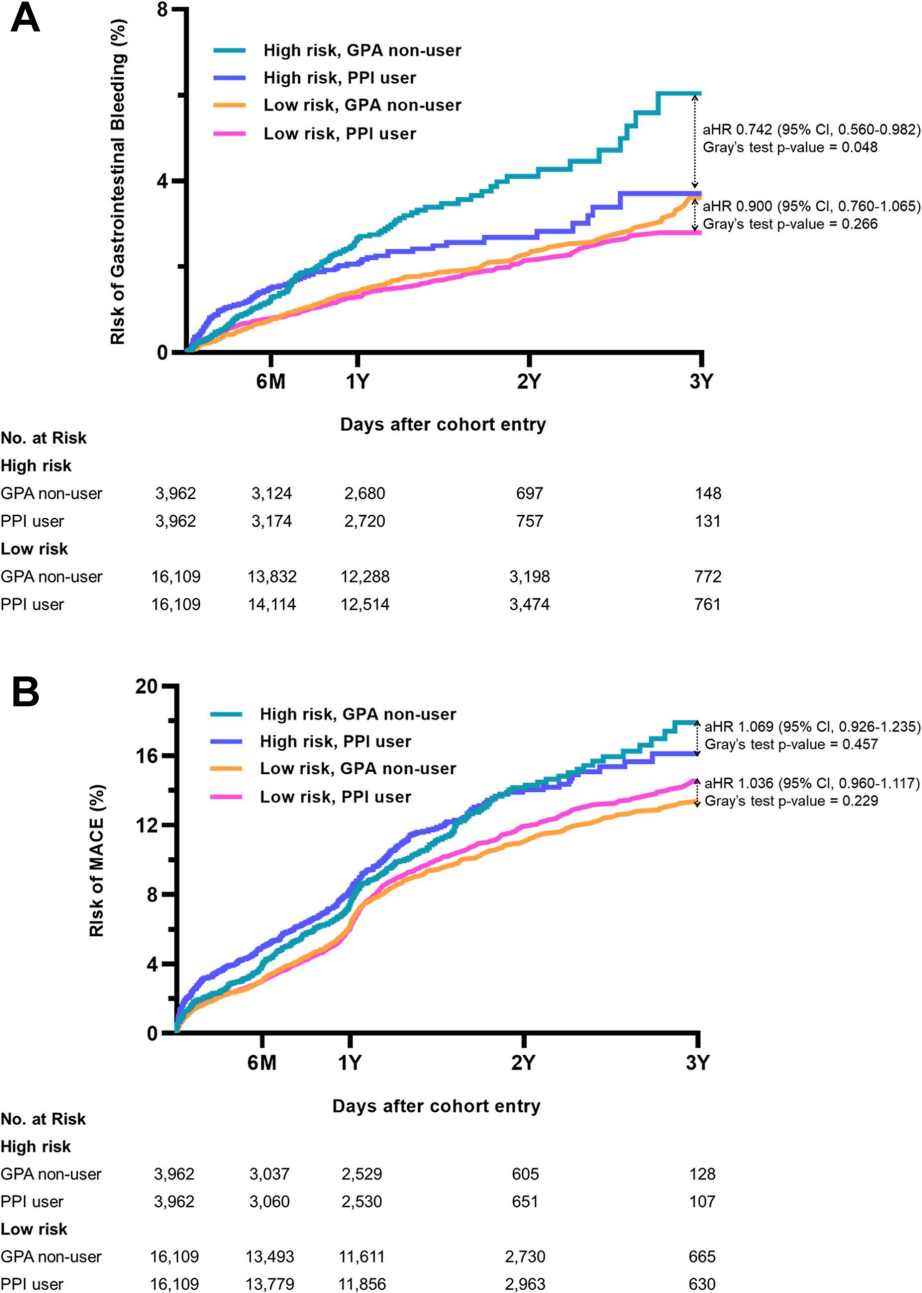
Cumulative incidence functions for major endpoints stratified by risk group and proton pump inhibitor use. **A** Cumulative incidence of GI bleeding: GPA non-user vs. PPI user; **B** cumulative incidence of MACE: GPA non-user vs. PPI user. *aHR* adjusted hazard ratio; *CI* confidence interval; *GI* gastrointestinal; *GPA* gastroprotective agents; *MACE* major adverse cardiovascular event; *PPI* proton pump inhibitor

In the high-risk group, PPI use was statistically associated with a lower GI bleeding incidence rate (1.72 vs. 2.29 per 100 person-years [PY], *p* = 0.044) (Table 2) and a reduced 3-year cumulative incidence (3.7% vs. 6.0%, *p* = 0.048) compared to GPA non-use (Fig. 3A). Multivariable analysis indicated that PPI use was associated with a 25.8% reduction in GI bleeding risk relative to GPA non-users (aHR 0.74, 95% CI 0.56–0.98). In contrast, no significant differences in MACE were observed (Table 2, Fig. 3B). In the high-risk group, age ≥ 75 years and a history of CKD were independent risk factors for GI bleeding (Online Resource Table 7). To support interpretability, we confirmed treatment consistency over time, with GPA group composition remaining stable during follow-up (Online Resource Table 8).

**Table 2:** Clinical outcomes associated with proton pump inhibitor use: incidence and adjusted hazard ratios in high- and low-risk patients

Clinical endpoint	Incidence rate per 100 person-years			Adjusted HR (95% CI)^a^
	GPA non-user (95% CI)	PPI user (95% CI)	*p*-value	
High risk				
GI bleeding	2.29 (1.89, 2.75)	1.72 (1.38, 2.12)	0.044	0.74 (0.56, 0.98)
MACE	7.74 (6.97, 8.58)	8.14 (7.35, 8.99)	0.492	1.07 (0.93, 1.24)
Low risk				
GI bleeding	1.28 (1.14, 1.44)	1.15 (1.02, 1.30)	0.228	0.90 (0.76, 1.07)
MACE	6.25 (5.91, 6.60)	6.51 (6.17, 6.86)	0.286	1.04 (0.96, 1.12)

GPA non-user served as a reference

^a^Adjusted by age ≥ 75 years, sex, dual antiplatelet therapy combination, cirrhosis with portal hypertension, chronic kidney disease, diabetes mellitus, dyspepsia, heart failure, hemorrhagic stroke, corticosteroid, and selective serotonin reuptake inhibitor

*CI* confidence interval, *GI* gastrointestinal, *GPA* gastroprotective agents, *HR* hazard ratio, *MACE* major adverse cardiovascular event, *PPI* proton pump inhibitor

### Impact of PPI Use on GI Bleeding and MACE Outcomes in Low-Risk Patients

In the low-risk group, PPI use was not significantly associated with GI bleeding risk. Incidence rates were similar between PPI users and GPA non-users (1.15 vs. 1.28/100 PY, *p* = 0.228) (Table 2), and 3-year cumulative incidence showed no significant difference (3.6% vs. 2.8%, *p* = 0.266) (Fig. 3A). Multivariable analysis found no significant evidence of a meaningful reduction in GI bleeding risk for PPI (aHR 0.90, 95% CI 0.76–1.07) in the low-risk cohort. MACE rates also showed no significant difference between PPI users and GPA non-users (Table 2, Fig. 3B). In the low-risk group, female sex and DM were identified as independent GI bleeding risk factors (Online Resource Table 7), and GPA cohort composition also remained stable over time (Online Resource Table 8).

### DAPT Subgroup Analysis: GI Bleeding and MACE Outcomes

In the high-risk group, PPI use with the AT combination was associated with lower GI bleeding risk compared to GPA non-use (aHR 0.55, 95% CI 0.32–0.94), while no significant reductions were observed in PPI users receiving AC or AP (Table 3). MACE outcomes did not differ substantially between PPI users and GPA non-users across all DAPT combinations in the high-risk group. In the low-risk group, PPI use did not significantly reduce GI bleeding risk for any DAPT combination, nor did it significantly affect MACE incidence (Table 4).

**Table 3:** Incidence and adjusted hazard ratios by dual antiplatelet therapy combination and proton pump inhibitor type in the high-risk group

			GI bleeding			MACE		
			Incidence rate per 100 person-years	*p*-value	Adjusted HR (95% CI)^a^	Incidence rate per 100 person-years	*p*-value	Adjusted HR (95% CI)^a^
Aspirin + Clopidogrel								
GPA non-user			2.13 (1.67, 2.68)	Ref	Ref	7.94 (7.00, 8.97)	Ref	Ref
PPI user			2.01 (1.21, 3.13)	0.818	0.89 (0.63, 1.24)	7.14 (5.51, 9.11)	0.446	1.01 (0.85, 1.20)
Omeprazole/esmoeprazole			1.77 (1.30, 2.36)	0.329	0.96 (0.58, 1.60)	8.06 (6.98, 9.26)	0.874	0.95 (0.72, 1.26)
Other PPIs			1.84 (1.42, 2.33)	0.382	0.86 (0.60, 1.25)	7.82 (6.90, 8.82)	0.855	1.03 (0.85, 1.24)
Aspirin + Prasugrel								
GPA non-user			2.58 (0.95, 5.62)	Ref	Ref	7.33 (4.19, 11.90)	Ref	Ref
PPI user			0.57 (0.02, 3.19)	0.126	0.21 (0.02, 2.73)	7.43 (3.84, 12.99)	0.970	1.02 (0.48, 2.19)
Aspirin + Ticagrelor								
GPA non-user			2.66 (1.85, 3.69)	Ref	Ref	7.29 (5.87, 8.95)	Ref	Ref
PPI user			1.57 (0.97, 2.39)	0.053	0.55 (0.32, 0.94)	9.12 (7.51, 10.98)	0.112	1.27 (0.96, 1.67)

GPA non-user served as a reference

^a^Adjusted by age ≥ 75 years, chronic kidney disease, diabetes mellitus, dyspepsia, heart failure, and corticosteroid

*CI* confidence interval, *GI* gastrointestinal, *GPA* gastroprotective agents, *HR* hazard ratio, *MACE* major adverse cardiovascular event, *PPI* proton pump inhibitor

**Table 4:** Incidence and adjusted hazard ratios by dual antiplatelet therapy combination and proton pump inhibitor type in the low-risk group

	GI bleeding			MACE		
	Incidence rate per 100 person-years	*p*-value	Adjusted HR (95% CI)^a^	Incidence rate per 100 person-years	*p*-value	Adjusted HR (95% CI)^a^
Aspirin + Clopidogrel						
GPA non-user	1.10 (0.92, 1.31)	Ref	Ref	6.68 (6.20, 7.19)	Ref	Ref
PPI user	1.26 (0.92, 1.70)	0.427	1.02 (0.80, 1.30)	7.16 (6.26, 8.15)	0.366	1.01 (0.91, 1.11)
Omeprazole/esmoeprazole	1.07 (0.87, 1.31)	0.866	1.16 (0.82, 1.64)	6.50 (5.97, 7.06)	0.619	1.08 (0.93, 1.25)
Other PPIs	1.13 (0.95, 1.33)	0.842	0.97 (0.74, 1.26)	6.68 (6.22, 7.16)	0.988	0.98 (0.88, 1.09)
Aspirin + Prasugrel						
GPA non-user	1.74 (1.18, 2.47)	Ref	Ref	4.83 (3.84, 5.99)	Ref	Ref
PPI user	1.27 (0.78, 1.93)	0.256	0.70 (0.40, 1.23)	5.96 (4.81, 7.30)	0.164	1.21 (0.89, 1.63)
Aspirin + Ticagrelor						
GPA non-user	1.43 (1.19, 1.70)	Ref	Ref	5.96 (5.45, 6.52)	Ref	Ref
PPI user	1.17 (0.96, 1.43)	0.144	0.83 (0.64, 1.08)	6.37 (5.84, 6.94)	0.291	1.06 (0.94, 1.21)

GPA non-user served as a reference

^a^Adjusted by age ≥ 75 years, chronic kidney disease, diabetes mellitus, dyspepsia, heart failure, and corticosteroid

*CI confidence interval, GI gastrointestinal, GPA gastroprotective agents, HR hazard ratio, MACE major adverse cardiovascular event, PPI proton pump inhibitor*

### Sensitivity Analysis

In the pooled cohort, PPI use was significantly associated with lower GI bleeding risk in high-risk patients (HR = 0.75, *p* = 0.042), but not in low-risk patients (HR = 0.89, *p* = 0.191) (Online Resource Table 9). Effect sizes were consistent with the stratified analysis, although the interaction term was not statistically significant (*p* = 0.288). A pooled estimate was not reported, as it may obscure subgroup-specific effects [43]. For MACE, no significant associations with PPI use were observed in either risk group, and the interaction was also non-significant (*p* = 0.794).

## Discussion

In this study, we applied the mARC-HBR criteria, a modified version of the validated ARC-HBR criteria tailored to claims data, to stratify bleeding risk, and compared its GI bleeding prediction performance with the existing AHA criteria. The mARC-HBR criteria identified a significantly higher proportion of high-risk patients, approximately three times more than the AHA criteria, and these patients exhibited markedly higher actual GI bleeding incidence. By including short-term high-risk indicators, such as recent bleeding events or major surgeries, and over 10 detailed clinical characteristics, the mARC-HBR criteria demonstrated better sensitivity (38.9% vs. 11.1%, *p* < 0.001) and captured more true-positive cases, albeit with slightly lower specificity (74.2% vs. 91.1%, *p* < 0.001), compared to the AHA criteria. While the mARC-HBR criteria’s broader risk stratification approach increases the likelihood of overtreatment, they provide a more comprehensive framework for identifying high-risk patients, making it a valuable tool for tailored GI bleeding prevention. Previous studies have supported the utility of the ARC-HBR criteria for predicting bleeding risk, highlighting their robust performance despite the complexity of the parameters [14, 22, 23]. In contrast, the simpler AHA criteria, with their narrower focus, often failed to classify patients with current high-risk conditions, underestimating the true bleeding risk. Overall, the mARC-HBR criteria offer an improved predictive approach but require complementary clinical judgment to optimize treatment decisions and minimize unnecessary interventions.

In each bleeding risk group, the analysis of GI bleeding and MACE incidence showed that PPI co-therapy was statistically associated with a lower risk of GI bleeding in the high-risk group, aligning with current guidelines and previous studies [11]. Specifically, PPI use was associated with a 25.8% reduction in GI bleeding risk compared to GPA non-users in the high-risk group, supporting its clinical benefit. However, this study did not evaluate other potential risks of PPI use, such as fractures or *Clostridium difficile* infections [45, 46]. Therefore, prescribing PPI requires careful consideration of each patient’s clinical context.

Notably, this study found no statistically significant protective effect of PPI therapy against GI bleeding in the low-risk group. While the impact of GPA use in low-risk groups has been underexplored, available literature presents inconsistent findings [14, 17]. For instance, a Chinese study reported a significantly higher GI bleeding rate in low-risk acute MI patients taking PPIs compared to GPA non-users (adjusted odds ratio 5.57, 95% CI 2.90–10.70), even after adjusting for 19 variables [17]. In contrast, a recent Korean study by Baik et al. observed a 46% reduction in GI bleeding requiring red blood cell transfusion among low-risk MI patients using PPIs (HR 0.54, 95% CI 0.43–0.68) [14]. Our study differs from Baik et al.’s in several key aspects. While both used Korean cohorts, we employed the ARC-HBR criteria for risk stratification, whereas Baik et al. used the AHA criteria, resulting in only 6.4% of their cohort being classified as high-risk [14]. This difference in stratification criteria means that a significant proportion of patients classified as low-risk in Baik et al.’s study may have been reclassified as high-risk under the ARC-HBR criteria, potentially affecting their results. Additionally, our study’s extended 3-year follow-up enabled a more robust evaluation of long-term GI bleeding risk compared to the 1-year follow-up in Baik et al.’s study.

This study further confirmed that PPI use does not adversely affect MACE incidence in patients receiving clopidogrel. Previous studies have raised concerns about potential interactions between PPIs, particularly omeprazole and esomeprazole, and clopidogrel due to CYP2C19 inhibition, which could impact clopidogrel activation and increase cardiovascular event risk [9]. However, our findings align with recent studies indicating that these interactions may have limited clinical relevance in real-world settings [47] and underscores the cardiovascular safety profile of PPIs in GI protection.

Sensitivity analyses supported the main findings, showing a consistent association between PPI use and lower GI bleeding risk in high-risk patients. However, the non-significant interaction terms suggest that the observed differential effect by bleeding risk status should be interpreted with caution. While pooled analysis improves statistical power, stratified evaluation was prioritized to maintain clinical relevance given distinct bleeding risk profiles [43]. No increased MACE risk was observed in either group.

The subgroup analysis of GI bleeding prevention by DAPT combination revealed that in high-risk patients, PPI use with the AT regimen was significantly associated with a lower GI bleeding risk (aHR 0.55, 95% CI 0.32–0.94), suggesting potential benefit for patients on ticagrelor. A similar, though statistically non-significant, trend was seen in the high-risk AP and AC groups, with the small AP sample size potentially affecting the detection of significance. In contrast, PPI use in the low-risk group was not associated with a significant reduction in GI bleeding risk across any DAPT combination, underscoring that routine PPI application may have limited clinical benefit in low-risk patients. These results support a targeted approach to PPI use, prioritizing high-risk patients, especially those on potent antiplatelet regimens like ticagrelor.

Notably, however, this subgroup analysis showed numerically higher MACE incidence among PPI users—27% in high-risk patients on AT, and 21% in low-risk patients on AP—though not statistically significant. While PPIs are not known to alter the pharmacokinetics of these agents [48], unmeasured confounding, including unmeasured cardiovascular risk profiles, differential disease severity, or potential long-term cardiovascular effects of PPI use [49] may have contributed. These findings should be interpreted cautiously, and further investigation is warranted to clarify whether underlying pharmacodynamic interactions or residual confounding may contribute to the observed trends in specific DAPT contexts.

The current study did not directly assess the appropriate duration of PPI use. While our findings support an association between continued PPI use and reduced GI bleeding risk in high-risk patients, decisions about long-term PPI use—particularly in those with remote GI bleeding history—should be individualized. Further prospective studies are warranted to guide duration of therapy.

This study offers several strengths that enhance its clinical applicability. It is the first to adapt the validated ARC-HBR criteria, designed for bleeding risk assessment in Asian populations, into a modified form (mARC-HBR) using claims data. This adaptation enabled the evaluation of GPAs and the identification of high-risk patients for GI bleeding with criteria more suited to Asian populations. Unlike the AHA criteria, which were developed based on Western populations and less reflective of the distinct bleeding characteristics of Asian patients [14, 17], the ARC-HBR-based approach better aligned with actual GI bleeding incidence, highlighting its applicability to Korean patients. Additionally, this study provides the first clinical evidence of PPIs preventing GI bleeding in AT users and offers a detailed comparison of GPA impact across common DAPT regimens—AC, AP, and AT. This analysis provides valuable insights into regimen-specific GI prophylaxis strategies. Finally, this study leveraged comprehensive nationwide claims data, providing objective real-world insights that enhance the generalizability of findings and support broader clinical applicability.

Despite these strengths, this study has several limitations. First, the ARC-HBR criteria were modified due to claims data constraints, limiting the interpretation of the results to the mARC-HBR criteria rather than the full ARC-HBR criteria. Further validation using detailed datasets, such as electronic medical records (EMRs), is necessary. Second, anticoagulant users were excluded to reduce variability from heterogeneous bleeding and thrombotic risks [32], but this exclusion oversimplified the application of the AHA criteria and omitted a clinically significant population. Third, the retrospective design precludes causal inference. While a statistically significant association was observed in high-risk patients, this result warrants cautious interpretation due to residual confounding and lack of randomization. Fourth, findings from Korean patients may limit generalizability of our results to other Asian or non-Asian populations; further validation in diverse populations is needed. Fifth, the reliance on claims data may not have accurately captured conditions like dyspepsia and lacked clinical details such as medication adherence, prescription rationale, and over-the-counter medication use [31]. *Helicobacter pylori* status was also unavailable, potentially leading to an oversimplified application of AHA risk criteria. Sixth, the analysis did not distinguish between oral and injectable PPI formulations, potentially including intensive care unit patients receiving intravenous PPIs for stress ulcer prophylaxis—whose clinical characteristics and bleeding risks differ significantly [50]. Lastly, the study focused on three DAPT combinations (AC, AP, AT), limiting generalizability to other regimens.

## Conclusions

In conclusion, this study is the first to adapt and apply the mARC-HBR criteria, a modified version of the ARC-HBR framework, to claims data for identifying high-risk GI bleeding patients in Korean population. By incorporating recent risk factors and demonstrating higher sensitivity—while maintaining reasonable specificity—compared to the AHA criteria, the mARC-HBR criteria provide valuable insights for optimizing GI bleeding prevention and advancing personalized treatment strategies for Korean patients. This study observed a statistically significant association between PPI use and reduced GI bleeding risk in high-risk patients, especially on potent DAPT regimens, such as ticagrelor, aligning with current guidelines, while no such association was found in low-risk patients. These findings were consistent across sensitivity analyses. Future research should validate these findings in diverse populations, explore real-word applicability using EMR data, and refine PPI dosing and duration.

## Supplementary Information

Below is the link to the electronic supplementary material.

ESM 1 (DOCX 52.2 KB)

## Data Availability

License-restricted Korean HIRA data cannot be shared publicly to meet ethical standards; analyzed data are provided in the article and Online Resources files.
