# Supplementary material for: A Novel Approach to Gastrointestinal Bleeding Risk Stratification and Proton Pump Inhibitor Effectiveness in Patients with Acute Coronary Syndrome on Dual Antiplatelet Therapy: A Nationwide Retrospective Cohort Study
Source: Cardiovasc Drugs Ther. 2025 Apr 26;40(1):263–76. doi: 10.1007/s10557-025-07702-4 (PMC12872704; doi:10.1007/s10557-025-07702-4)
Supplement: Supplementary file 1 — (DOCX 52.2 KB) [file 10557_2025_7702_MOESM1_ESM.docx]

**Online Resources**

**Article title: A Novel Approach to Gastrointestinal Bleeding Risk Stratification and Proton Pump Inhibitor Effectiveness in Patients with Acute Coronary Syndrome on Dual Antiplatelet Therapy: A Nationwide Retrospective Cohort Study**

**Journal name:** Cardiovascular Drugs and Therapy

**Author names:** Mee Yeon Lee, Kyu-Nam Heo, Jaekyu Shin, Ju-Yeun Lee

**Corresponding Author:** Professor Ju-Yeun Lee

College of Pharmacy and Research Institute of Pharmaceutical Sciences, Seoul National University, 1, Gwanak-ro, Gwanak-gu, Seoul 08826, Republic of Korea

Telephone: +82-2-3668-7472

E-mail address: jypharm@snu.ac.kr

**Online Resource Table 1. Operational definitions and KCD codes for high bleeding risk factors in mARC-HBR**

| **Risk Factors** | | **Operational Definition** | **Codes** |
| --- | --- | --- | --- |
| **Major Risk Factors** | | | |
|  | Anticipated longterm oral anticoagulation | Patients with a primary or secondary disgnosis code for atrial fibrillation within the 12 months prior to the index date, who underwent PCI during the same hospitalization period. These patients were excluded from the analysis. | Atrial fibrillation: I48, I480, I481, I482, I489, I4890 PCI (procedure codes): M6551, M6552, M6561, M6562, M6563, M6564, M6571, M6572 |
|  | Estimated GFR <30 mL/min | Patients with a primary or secondary disgnosis code for dialysis, end-stage renal disease (ESRD), or ESRD associated with diabetes or hypertensive/congenital renal failure within the 12 months prior to the index date, or those using potassium binder (sodium polystyrene sulfonate), erythropoietin, or phosphate binders (calcium acetate, calcium carbonate, sevelamer carbonate) with kidney-related diagnosis. | Dialysis (Treatment codes): O7020, O7021, O707, O9991 ESRD: N18, N184, N185, N189, N19, Z49, Z490, Z491, Z492, Z992 Diabetes with end-stage renal disease or hypertensive or congenital renal failure: E1022, E1122, E1222, E1322, E1422, I120, I131, I132, P960 |
|  | Haemoglobin <11 g/dL | Not applicable due to the unavailability of laboratory data. | |
|  | Spontaneous bleed requiring hospitalization or transfusion within six months or recurrent bleed | Transfusion procedure code exists within six months prior to the index date, without the need for a primary diagnosis code during hospitalization, or bleeding primary diagnosis code requring hospitalization. | Whole blood and red blood cell transfusion codes: X1001, X1002, X2021, X2022, X2031, X2032, X2091, X2092, X2111, X2112, X2131, X2132 Platelet transfusion codes: X2071, X2072, X2081, X2082, X2121, X2122, X2516 Bleeding: I850, I8500, I8501, I983, K2211, K228, K250, K2500, K2501, K252, K2521, K2540, K2541, K256, K2561, K260, K262, K264, K266, K270, K272, K274, K276, K280, K282, K284, K286, K290, K3181, K5521, K5701, K5703, K5711, K5713, K5721, K5723, K5731, K5733, K5741, K5743, K5751, K5753, K5781, K5783, K5791, K5793, K661, K920, K921, K922, K625, K226, I60, I600, I601, I602, I603, I604, I605, I606, I607, I608, I609, I61, I610, I611, I612, I613, I614, I615, I616, I618, I619, I62, I620, I621, I629, S064, S0640, S0641, S065, S0650, S0651, S066, S0660, S0661, D683, D684, D688, D689, D69, D690, D698, D699, H052, H113, H210, H313, H356, H431, H450, H470, H6031, H922, I312, J942, M250, M2500, M2501, M2502, M2503, M2504, M2505, M2506, M2507, M2508, M2509, N02, N020, N021, N022, N023, N024, N025, N026, N027, N028, N029, N402, N403, N421, N836, N837, N923, N924, N926, N93, N930, N938, N939, N950, R04, R040, R041, R042, R048, R049, R233, R31, R310, R311, R318, R58, T792, T810 |
|  | Platelet count <100 × 10^9^ per litre | Not applicable due to the unavailability of laboratory data. | |
|  | Bleeding diathesis or cirrhosis with portal hypertension | Both cirrhosis and portal hypertension primary or secondary diagnosis codes are present within 12 months prior to the index date. Bleeding diathesis is not applicable. | Cirrhosis: Liver cirrhosis (K703, K74, K740, K741, K742, K743, K744, K745, K746, K761), Esophageal varix (I85, I850, I859, I982, I983, K22), hepatic encephalopathy (K72), hepatorenal syndrome (K767) Portal hypertension: Esophageal varix (I85, I850, I859, I982, I983, K22), hepatic encephalopathy (K72), hepatorenal syndrome (K767), ascites (K66, K7031, R18, R190, R60), spontaneous bacterial peritonitis (K65, without K67 co-diagnosis), hepatopulmonary syndrome and portopulmonary hypertension (I272), portal vein thrombosis (I81) |
|  | Active malignancy (excluding non-melanoma skin cancer) within 12 months | Primary diagnosis code for cancer within 12 months prior to the index date. | Mild cancer: C00, C01, C02, C03, C04, C05, C06, C07, C08, C09, C10, C11, C12, C13, C14, C15, C16, C17, C18, C19, C20, C21, C22, C23, C24, C25, C26, C30, C31, C32, C33, C34, C37, C38, C39, C40, C41, C45, C46, C47, C48, C49, C50, C51, C52, C53, C54, C55, C56, C57, C58, C60, C61, C62, C63, C64, C65, C66, C67, C68, C69, C70, C71, C72, C73, C74, C75, C76, C7A, C7B, C81, C82, C83, C84, C85, C86, C88, C90, C91, C92, C93, C94, C95, C96 Metastatic cancer: C77, C78, C79, C80 |
|  | Previous spontaneous ICH | Primary or secondary diagnosis code for intracerebral hemorrhage or its sequelae within 12 months prior to the index date. | Intracerebral haemorrhage: I61 Sequelae of intracerebral haemorrhage: I691 |
|  | Previous traumatic ICH within the past 12 months | Primary or secondary diagnosis code for traumatic ICH within 12 months prior to the index date. | Traumatic ICH: S063, S0630, S0631 |
|  | Presence of a bAVM | Primary or secondary diagnosis code for bAVM within 12 months prior to the index date were identified, but no cases were found. | Arteriovenous malformation of precerebral vessels: Q280 Arteriovenous malformation of cerebral vessels: Q282 |
|  | Moderate or severe ischaemic stroke within six months | Patients with a primary diagnosis of ischemic stroke or its sequelae within 12 months prior to the index date, and a primary diagnosis of ischemic stroke accompanied by hospitalization within the past six months. | Ischemic stroke: I630, I631, I632, I633, I634, I635, I636, I638, I639, I64, I678 Sequelae of cerebral infarction: I693 |
|  | Non-deferrable major surgery on DAPT | Not applicable due to the unavailability of clinical data. | |
|  | Recent major surgery or trauma within 30 days | Patients with a primary or secondary diagnosis code for trauma within one month prior to the index date, with no trauma diagnosis between 12 months and one month prior to the index date, or those with a procedure code for major surgery within one month prior to the index date. Major surgery was defined by the surgery to the major bleeding sites: Pulmonary bleeding (trachea, bronchi, and lungs), pericardial bleeding (cardiovascular system), retroperitoneal bleeding (spleen and lymph nodes, peritoneum and retroperitoneum, endocrine organs, liver, gallbladder and bile ducts, pancreas, organ transplantation), gastrointestinal bleeding (esophagus, stomach, intestines, mesentery, hernia, rectum and anus), genitourinary bleeding (urinary tract, male reproductive system, female reproductive system, pregnancy and childbirth), and intracranial hemorrhage (nervous system). All gastrointestinal endoscopic procedures were excluded. | Trauma: S00, S01, S02, S03, S04, S05, S06, S07, S08, S09, S10, S11, S12, S13, S14, S15, S16, S17, S18, S19, S20, S21, S22, S23, S24, S25, S26, S27, S28, S29, S30, S31, S32, S33, S34, S35, S36, S37, S38, S39, S40, S41, S42, S43, S44, S45, S46, S47, S48, S49, S50, S51, S52, S53, S54, S55, S56, S57, S58, S59, S60, S61, S62, S63, S64, S65, S66, S67, S68, S69, S70, S71, S72, S73, S74, S75, S76, S77, S78, S79, S80, S81, S82, S83, S84, S85, S86, S87, S88, S89, S90, S91, S92, S93, S94, S95, S96, S97, S98, S99,-S99, T00, T01, T02, T03, T04, T05, T06, T07, T79, G54, G56, G57, G935, G936, H210, H261, H922, I31, I770, I772, J43, J93, J94, J982, K223, K661, M125, M483, M610 Major surgery (procedure codes): O1401, O1403, O1404, O1405, O1410, O1421, O1422, O1423, O1424, O1431, O1432, O1440, O1450, O1460, O1471, OA631, OB631, OA632, OB632, OA633, OB633, OA634, OB634, OA635, OB635, OA636, OB636, OA637, OB637, OA638, OB638, OA639, OB639, O1635, O1641, OA641, O1640, OA640, O1648, OA648, O1649, OA649, O1647, OA647, O1643, O1644, O0175, O0176, O0161, O0162, O0163, O0164, O0165, O0166, O0167, O0168, O0169, O0170, O0171, O0172, O0173, O0174, O1645, O1646, OB641, OB642, OB643, O1650, O1651, O1654, OA654, O1655, OA655, O1656, OA656, O1657, O1658, O1659, O2651, O2652, O2653, OA651, O2650, O1660, O1671, O1672, O1680, O1690, O1701, O1702, O1703, O1704, O1705, O1710, O1711, O1721, O1723, O1722, O1730, O1740, O1750, O1760, O1770, O1781, O1784, O1782, O1783, O1791, O1792, O1793, O1797, O1794, O1795, O1796, O1798, O1799, O1810, O1821, O1822, O1823, O1824, O1825, O1826, O1830, O1840, O1841, O1842, O1843, O1844, O1851, O1852, O1861, O1873, O1874, O1875, O1878, O1879, O0881, O0882, O0883, O0884, O0885, O1890, O1895, O1901, O1902, O1907, O1903, O1904, O1910, O1921, O1922, O1931, O1932, O1935, O1940, O1950, O1960, O1970, O1981, O1982, O0203, O0204, O0230, O0205, O0206, O0207, O0208, O0209, O0210, O2009, O2004, O0241, O0242, O0243, O2006, O2007, O0211, O0212, O0213, O0214, O0219, O0220, O0221, O0222, O2211, O2212, O2213, O2214, O2215, O2216, O2030, O2011, O2012, O2081, O2082, O2084, O2083, O2031, O2021, O2022, O2032, O2033, O0223, O0224, O2034, O2037, O2038, O2039, O2035, O2040, O2045, O2053, O2054, O2055, O2056, O2057, O2058, O0218, O2059, O0260, O0261, O0262, O0263, O0264, O0265, O0266, O0267, O0226, O0227, O2066, O2064, O2067, O2065, O2068, O2071, O2072, O2073, O2074, O0277, O0278, O0279, O0280, O0281, O0282, P2081, P2082, P2091, P2093, P2094, P2102, P2103, P2106, P2107, 2121, P2122, P2123, P2124, P2131, P2133, P2134, P2135, P2136, P2137, P2138, P2141, P2142, Q2346, Q2347, Q2348, Q2365, Q2366, Q2367, Q2368, Q2369, Q2390, Q2391, Q2392, Q2401, Q2402, Q2403, QA421, QA422, QA423, QA424, QA425, QA426, Q2423, Q2424, Q2431, Q2430, Q2433, Q2432, Q2438, Q2440, Q2445, Q2450, Q2482, Q2501, Q2502, QA536, Q2533, Q2536, Q2534, Q2537, Q2550, Q2551, Q2552, Q2561, Q2562, Q2571, Q2572, Q2573, Q0259, Q2594, Q0251, Q0252, Q0253, Q0254, Q0255, Q0256, Q0257, Q0258, Q2598, Q2601, Q2640, Q2651, Q2650, QA671, Q2671, Q1261, Q1262, QA672, Q2672, QA673, Q2673, QA679, Q2679, Q2676, Q2687, Q2688, Q2680, Q2691, Q2692, Q2693, Q2690, Q2710, Q2761, Q2762, Q2773, Q2774, Q2775, Q2771, Q2781, Q2791, Q2792, Q2793, Q2794, Q2796, Q2797, Q2798, Q2801, Q2802, Q2803, Q2804, Q2810, Q2831, Q2832, Q2841, Q2842, Q2861, Q2862, Q2863, Q2871, Q2872, Q2875, Q2891, Q2890, Q2892, Q2893, Q2901, Q2910, Q2927, QA921, QA922, Q2922, Q0292, QA928, Q2928, QA923, Q2923, QA924, Q2924, QA925, Q2925, QA926, Q2926, Q2933, Q2934, Q2935, Q2936, Q2937, Q2938, Q2981, Q2982, Q2983, Q2984, Q2985, Q2991, Q2992, R3151, R3152, R3153, R3154, R3156, R3170, R3180, R3220, R3231, R3241, R3251, R3271, R3274, R3275, R3273, R3290, R3307, R3312, R3330, R3433, R3432, R3470, R3481, R3482, R3550, R3590, R3755, R3756, R3791, R3792, R3801, R3802, R3851, R3852, R3853, R3861, R3862, R3891, R3901, R3902, R3930, R3950, RZ512, R3960, R4066, R4067, R4068, R4070, R4071, R4073, R4072, R4074, R4124, R4127, R4123, R4143, R4144, R4147, R4148, R4149, R4140, R4154, R4155, R4157, R4221, R4223, R4224, R4250, RA431, RA432, RA433, RA434, RA311, RA312, RA313, RA314, RA315, RA316, RA317, RA318, R4351, R4353, R4356, R4358, R4421, R4423, R4427, R4424, R4428, R4425, R4426, R4430, R4507, R4508, R4509, R4510, R5001, R5002, R4517, R4518, R4514, R4519, R4520, R4516, P4541, P4542, P4543, P4545, P4561, P4565, P4571, P4572, P4581, P4582, S4593, S4594, S4595, S4596, S4605, S4606, S4607, S4601, S4602, S4603, S4604, S4611, S4612, S4613, S4615, S4616, S4621, S4622, S4625, S4634, S4635, S4636, S4637, S4638, S4639, S4641, S4642, S4653, S4654, S4655, S4656, S4657, S4658, S4661, S4662, S4670, S4671, S4681, S4682, S4684, S4683, S4685, S6691, S6692, S6693, S6694, S6695, S6696, S4694, S4695, S4696, S4704, S4705, S4706, S4707, S4708, S4709, S4711, S4712, S4713, S4714, S4715, S4721, S4722, S4723, S4724, S4731, S4732, S0435, S0431, S0432, S0436, S4733, S0433, S4735, S4736, S4737, S0434, S0471, S0472, S0477, S0478, S0474, S0475, S0476, S4730, S4745, S4746, S4747, S4748, S4741, S4742, S4743, S4744, S4756, S4757, S4758, S4760, S4771, S4772, S4780, S4796, S4792, S4793, S4794, S4797, S0479, S4798, S4799, S4801, S4802, S4803, S4805, S4821, S4822, S4827, S4823, S4824, S4831, S4832, S4833, S4834, S4835, S4836, S4841, S4842, S4843, S844, S4845, S4846, S4847, S4848, S4861, S4862, S4863, S4864, S4865, S4866, S4867, S4868, SY621, SY622, SY633, SY634, SY635, SY636, SY637, SY638, SY639, SY625, SY626, Q7221, Q7222, Q7225, Q7223, Q7224, Q7230, Q7240, Q7250, Q7260, Q7270, Q7282, Q7283, Q7332, Q7341, Q7342, Q7351, Q7352, Q7371, Q7372, Q7380, Q7400, Q7410, Q7520, Q7541, Q7542, Q7543, Q7550, Q7561, Q7562, Q7563, Q7564, Q7567, Q7565, Q7566, Q7571, Q7572, Q7591, Q7592, R3272, R3280, Q8030, Q8031, Q8032, Q8034, Q8035, Q8036, Q8037, Q8040, Q8041, Q8042, Q8043, Q8044, Q8045, Q8046, Q8047, Q8048, Q8049, Q8050, Q8140, Q8141, Q8142, Q8143, Q8144, Q8145, Q8146, Q8147, Q8148, Q8149, Q8150, Q8051, Q8052, Q8061, Q8062, Q8070, Q8080, Q8091, Q8092, Q8101, Q8102, Q8103, Q8111, Q8112, Q8121, Q8122, Q8123, Q8130, Q8131 |
| **Minor Risk Factors** | | | |
|  | Age ≥75 years | Patients aged more than or eqaul to 75 years. |  |
|  | Estimated GFR 30–59 mL/min | Not applicable due to the unavailability of laboratory data. | |
|  | Haemoglobin 11–12.9 g/dL for men and 11–11.9 g/dL for women | Not applicable due to the unavailability of laboratory data. | |
|  | Spontaneous bleed requiring hospitalization or transfusion within 12 months not meeting major feature | Transfusion procedure code exists between 12 months and six months prior to the index date, without the need for a primary diagnosis code during hospitalization, or bleeding primary diagnosis code requring hospitalization. | Whole blood and red blood cell transfusion codes: X1001, X1002, X2021, X2022, X2031, X2032, X2091, X2092, X2111, X2112, X2131, X2132 Platelet transfusion codes: X2071, X2072, X2081, X2082, X2121, X2122, X2516 Bleeding: I850, I8500, I8501, I983, K2211, K228, K250, K2500, K2501, K252, K2521, K2540, K2541, K256, K2561, K260, K262, K264, K266, K270, K272, K274, K276, K280, K282, K284, K286, K290, K3181, K5521, K5701, K5703, K5711, K5713, K5721, K5723, K5731, K5733, K5741, K5743, K5751, K5753, K5781, K5783, K5791, K5793, K661, K920, K921, K922, K625, K226, I60, I600, I601, I602, I603, I604, I605, I606, I607, I608, I609, I61, I610, I611, I612, I613, I614, I615, I616, I618, I619, I62, I620, I621, I629, S064, S0640, S0641, S065, S0650, S0651, S066, S0660, S0661, D683, D684, D688, D689, D69, D690, D698, D699, H052, H113, H210, H313, H356, H431, H450, H470, H6031, H922, I312, J942, M250, M2500, M2501, M2502, M2503, M2504, M2505, M2506, M2507, M2508, M2509, N02, N020, N021, N022, N023, N024, N025, N026, N027, N028, N029, N402, N403, N421, N836, N837, N923, N924, N926, N93, N930, N938, N939, N950, R04, R040, R041, R042, R048, R049, R233, R31, R310, R311, R318, R58, T792, T810 |
|  | Chronic use of NSAIDs or steroids | If the total number of days prescribed for oral NSAIDs or steroids in the three months prior to the index date exceeds 35 days. |  |
|  | Any ischaemic stroke not meeting major feature | Not applicable due to the unavailability of clinical data. | |

*bAVM* brain arteriovenous malformation; *DAPT* dual antiplatelet therapy; *GFR* glomerular filtration rate; *ICH* intracerebral hemorrhage; *KCD* Korean standard classification of diseases; *NSAID* non-steroidal anti-inflammatory drug; *PCI* percutaneous coronary intervention

**Online Resource Table 2. STROBE Statement—Checklist of items that should be included in reports of cohort studies**

|  | Item No | Recommendation | Page No |
| --- | --- | --- | --- |
| **Title and abstract** | 1 | (*a*) Indicate the study’s design with a commonly used term in the title or the abstract | 1,2 |
|  |  | (*b*) Provide in the abstract an informative and balanced summary of what was done and what was found | 2 |
| Introduction | | | |
| Background/rationale | 2 | Explain the scientific background and rationale for the investigation being reported | 3,4 |
| Objectives | 3 | State specific objectives, including any prespecified hypotheses | 4 |
| Methods | | | |
| Study design | 4 | Present key elements of study design early in the paper | 5-8 |
| Setting | 5 | Describe the setting, locations, and relevant dates, including periods of recruitment, exposure, follow-up, and data collection | 5-8 |
| Participants | 6 | (*a*) Give the eligibility criteria, and the sources and methods of selection of participants. Describe methods of follow-up | 5-8 |
|  |  | (*b*) For matched studies, give matching criteria and number of exposed and unexposed | 6,8,20 |
| Variables | 7 | Clearly define all outcomes, exposures, predictors, potential confounders, and effect modifiers. Give diagnostic criteria, if applicable | 6,7 |
| Data sources/ measurement | 8* | For each variable of interest, give sources of data and details of methods of assessment (measurement). Describe comparability of assessment methods if there is more than one group | 5-8 |
| Bias | 9 | Describe any efforts to address potential sources of bias | 6-8 |
| Study size | 10 | Explain how the study size was arrived at | 5,6,20 |
| Quantitative variables | 11 | Explain how quantitative variables were handled in the analyses. If applicable, describe which groupings were chosen and why | 8 |
| Statistical methods | 12 | (*a*) Describe all statistical methods, including those used to control for confounding | 8 |
|  |  | (*b*) Describe any methods used to examine subgroups and interactions | 8 |
|  |  | (*c*) Explain how missing data were addressed | 5,6 |
|  |  | (*d*) If applicable, explain how loss to follow-up was addressed | 5,6 |
|  |  | (*e*) Describe any sensitivity analyses | 8 |
| Results | | |  |
| Participants | 13* | (a) Report numbers of individuals at each stage of study—eg numbers potentially eligible, examined for eligibility, confirmed eligible, included in the study, completing follow-up, and analysed | 8,9 |
|  |  | (b) Give reasons for non-participation at each stage | 5,9,20 |
|  |  | (c) Consider use of a flow diagram | 20 |
| Descriptive data | 14* | (a) Give characteristics of study participants (eg demographic, clinical, social) and information on exposures and potential confounders | 8,9 |
|  |  | (b) Indicate number of participants with missing data for each variable of interest | N/A (6) |
|  |  | (c) Summarise follow-up time (eg, average and total amount) | 6,7,10,11 |
| Outcome data | 15* | Report numbers of outcome events or summary measures over time | 9-11 |
| Main results | 16 | (*a*) Give unadjusted estimates and, if applicable, confounder-adjusted estimates and their precision (eg, 95% confidence interval). Make clear which confounders were adjusted for and why they were included | 10,11 |
|  |  | (*b*) Report category boundaries when continuous variables were categorized | 6,7 |
|  |  | (*c*) If relevant, consider translating estimates of relative risk into absolute risk for a meaningful time period | 9-11 |
| Other analyses | 17 | Report other analyses done—eg analyses of subgroups and interactions, and sensitivity analyses | 9-11 |
| Discussion |  |  |  |
| Key results | 18 | Summarise key results with reference to study objectives | 11-13 |
| Limitations | 19 | Discuss limitations of the study, taking into account sources of potential bias or imprecision. Discuss both direction and magnitude of any potential bias | 14,15 |
| Interpretation | 20 | Give a cautious overall interpretation of results considering objectives, limitations, multiplicity of analyses, results from similar studies, and other relevant evidence | 11-15 |
| Generalisability | 21 | Discuss the generalisability (external validity) of the study results | 14 |
| Other information |  |  |  |
| Funding | 22 | Give the source of funding and the role of the funders for the present study and, if applicable, for the original study on which the present article is based | 15 |

*Give information separately for exposed and unexposed groups.

**Online Resource Table 3. KCD codes used for outcomes and comorbidities**

| **Disease** | | **KCD Codes** |
| --- | --- | --- |
| **Outcome** | |  |
|  | GI bleeding | I850, I8500, I8501, I983, K2211, K228, K250, K2500, K2501, K252, K2521, K2540, K2541, K256, K2561, K260, K262, K264, K266, K270, K272, K274, K276, K280, K282, K284, K286, K290, K3181, K5521, K5701, K5703, K5711, K5713, K5721, K5723, K5731, K5733, K5741, K5743, K5751, K5753, K5781, K5783, K5791, K5793, K661, K920, K921, K922, K625, K226 |
|  | MACE | - AMI: I21, I210, I211, I212, I213, I214, I219 - Stroke: I60, I601, I602, I603, I604, I605, I606, I607, I608, I609, I61, I610, I611, I612, I613, I614, I615, I616, I618, I619, I62, I620, I621, I629, I63, I630, I6300, I6301, I6302, I6308, I6309, I631, I6310, I6311, I6312, I6318, I6319, I632, I6320, I6321, I6322, I6328, I6329, I633, I6330, I6331, I6332, I6333, I6338, I6339, I634, I6340, I6341, I6342, I6343, I6348, I6349, I635, I6350, I6351, I6352, I6353, I6358, I6359, I636, I638, I639, I64 - Cardiovascular death: I00, I01, I02, I05, I06, I07, I08, I09, I10, I11, I12, I13, I14, I15, I20, I21, I22, I23, I24, I25, I26, I27, I28, I30, I31, I32, I33, I34, I35, I36, I37, I38, I39, I40, I41, I42, I43, I44, I45, I46, I47, I48, I49, I50, I51, I52, I60, I61, I62, I63, I64, I65, I66, I67, I68, I69, I70, I71, I72, I73, I74, I75, I76, I77, I78, I79, I80, I81, I82, I83, I84, I85, I86, I87, I88, I89, I95, I96, I97, I98, I99 |
| **Comorbidities** | |  |
|  | Alcoholism | E244, F10, G312, G621, G721, I426, K292, K70, K860, O354, P043, Q860, T51, Y90, Z714 |
|  | Chronic kidney disease | N18, E0822, E0922, E1022, E1122, E1322, I12, I13, Z940, Z992 |
|  | Diabetes mellitus | E08, E09, E10, E11, E13 |
|  | Dyspepsia | K30, R1013 |
|  | Gastroesophageal reflux disease | K21, K221 |
|  | Heart failure | I50 |
|  | Hemorrhagic stroke | I60, I61 |
|  | Hypertension | I10, I11, I12, I13, I14, I15, O10, O11, O13, O16, P292 |
|  | Irritable bowel disease | K500, K501, K508, K509, K510, K512, K513, K514, K515, K518, K519 |
|  | Ischemic stroke | I6782, I63, I64 |
|  | Non-severe peptic ulcer disease | K25, K253, K257, K259, K26, K263, K267, K269, K27, K273, K277, K279, K28, K283, K287, K289, K633 |
|  | PCI insertion during hospitalization | (Procedure code) M6551, M6552, M6561, M6562, M6563, M6564, M6571, M6572 |
|  | Severe peptic ulcer disease | K250, K251, K252, K254, K255, K256, K260, K261, K262, K264, K265, K266, K270, K271, K272, K274, K275, K276, K280, K281, K282, K284, K285, K286 |
|  | Thrombocytopenia | D691, D693, D694, D6941, D6942, D6949, D695, D6951, D6959, D696 |
|  | Transient ischemic attack | G45 |

*AMI* acute myocardial infarction; *GI* gastrointestinal; *KCD* Korean standard classification of diseases; *MACE* major adverse cardiovascular event; *PCI* percutaneous coronary intervention

**Online Resource Table 4. Baseline characteristics of dual antiplatelet therapy users hospitalized for acute coronary syndrome by risk grade and proton pump inhibitor use (before propensity score matching)**

| **Characteristics** | | **High risk group (N=16,450)** | | | **Low risk group (N=46,089)** | | |
| --- | --- | --- | --- | --- | --- | --- | --- |
|  |  | **GPA Non-user (N=3,962)** | **PPI User (N=12,488)** | **p-value** | **GPA Non-user (N=16,109)** | **PPI User (N=29,980)** | **p-value** |
|  |  | **N(%)** | **N(%)** |  | **N(%)** | **N(%)** |  |
| **Age, years** | |  |  |  |  |  |  |
|  | <65 | 937 (23.7) | 2,189 (17.5) | <.001 | 7,254 (45.0) | 11,204 (37.4) | <.001 |
|  | 65–74 | 1,673 (42.2) | 4,741 (38.0) |  | 6,626 (41.1) | 13,337 (44.5) |  |
|  | 75–84 | 1,148 (29.0) | 4,535 (36.3) |  | 1,887 (11.7) | 4,563 (15.2) |  |
|  | ≥85 | 204 (5.2) | 1,023 (8.2) |  | 342 (2.1) | 876 (2.9) |  |
| **Sex, male** | | 2,871 (72.5) | 7,748 (62.0) | <.001 | 13,178 (81.8) | 22,265 (74.3) | <.001 |
| **Dual antiplatelet therapy types** | |  |  |  |  |  |  |
|  | Aspirin/clopidogrel | 2,833 (71.5) | 9,384 (75.1) | <.001 | 8,769 (54.4) | 17,164 (57.3) | <.001 |
|  | Aspirin/prasugrel | 151 (3.8) | 331 (2.7) |  | 1,197 (7.4) | 1,937 (6.5) |  |
|  | Aspirin/ticagrelor | 978 (24.7) | 2,773 (22.2) |  | 6,143 (38.1) | 10,879 (36.3) |  |
| **CCI score (mean ± SD)** | | 3.9 ± 2.2 | 4.0 ± 2.3 | <.001 | 2.0 ± 1.5 | 2.3 ± 1.6 | <.001 |
|  | 0–1 | 544 (13.7) | 1,501 (12.0) | <.001 | 6,615 (41.1) | 10,366 (34.6) | <.001 |
|  | 2–4 | 2,010 (50.7) | 6,241 (50.0) |  | 8,595 (53.4) | 16,915 (56.4) |  |
|  | ≥5 | 1,408 (35.5) | 4,746 (38.0) |  | 899 (5.6) | 2,699 (9.0) |  |
| **PCI insertion during hospitalization** | | 2,545 (64.2) | 7,783 (62.3) | 0.030 | 10,661 (66.2) | 19,992 (66.7) | 0.274 |
| **Comorbidities** | |  |  |  |  |  |  |
|  | Alcoholism | 76 (1.9) | 273 (2.2) | 0.308 | 210 (1.3) | 541 (1.8) | <.001 |
|  | Chronic kidney disease | 958 (24.2) | 2,304 (18.5) | <.001 | 84 (0.5) | 200 (0.7) | 0.057 |
|  | Diabetes mellitus | 1,712 (43.2) | 5,257 (42.1) | 0.216 | 4,401 (27.3) | 8,702 (29.0) | <.001 |
|  | Dyspepsia | 885 (22.3) | 3,712 (29.7) | <.001 | 3,110 (19.3) | 7,554 (25.2) | <.001 |
|  | Gastroesophageal reflux disease | 1,313 (33.1) | 5,923 (47.4) | <.001 | 4,578 (28.4) | 13,197 (44.0) | <.001 |
|  | Heart failure | 418 (10.6) | 1,449 (11.6) | 0.069 | 809 (5.0) | 1,769 (5.9) | <.001 |
|  | Hemorrhagic stroke | 61 (1.5) | 217 (1.7) | 0.399 | 18 (0.1) | 46 (0.2) | 0.252 |
|  | Hypertension | 2,519 (63.6) | 8,407 (67.3) | <.001 | 8,246 (51.2) | 16,348 (54.5) | <.001 |
|  | Irritable bowel disease | 14 (0.4) | 33 (0.3) | 0.360 | 29 (0.2) | 68 (0.2) | 0.296 |
|  | Ischemic stroke | 483 (12.2) | 1,964 (15.7) | <.001 | 462 (2.9) | 1,257 (4.2) | <.001 |
|  | Non-severe peptic ulcer disease | 625 (15.8) | 2,878 (23.1) | <.001 | 2,088 (13.0) | 5,565 (18.6) | <.001 |
|  | Severe peptic ulcer disease | 45 (1.1) | 321 (2.6) | <.001 | 74 (0.5) | 221 (0.7) | <.001 |
|  | Thrombocytopenia | 13 (0.3) | 51 (0.4) | 0.479 | 17 (0.1) | 38 (0.1) | 0.529 |
|  | Transient ischemic attack | 103 (2.6) | 403 (3.2) | 0.046 | 225 (1.4) | 595 (2.0) | <.001 |
| **Co-medications** | |  |  |  |  |  |  |
|  | Corticosteroid | 353 (8.9) | 2,128 (17.0) | <.001 | 1,096 (6.8) | 3,395 (11.3) | <.001 |
|  | COX2 inhibitor | 133 (3.4) | 984 (7.9) | <.001 | 115 (0.7) | 715 (2.4) | <.001 |
|  | Ketolorac | 35 (0.9) | 255 (2.0) | <.001 | 184 (1.1) | 444 (1.5) | 0.003 |
|  | Non-ketolorac traditional NSAID | 346 (8.7) | 2,463 (19.7) | <.001 | 913 (5.7) | 3,618 (12.1) | <.001 |
|  | Selective serotonin reuptake inhibitor | 144 (3.6) | 697 (5.6) | <.001 | 250 (1.6) | 811 (2.7) | <.001 |

Values are expressed as mean ± SD, or percentages.

*CCI* Charlson Comorbidity Index; *COX2* cyclooxygenase-2; *GPA* gastroprotective agents; *NSAID* non-steroidal anti-inflammatory drugs; *PCI* percutaneous coronary intervention; *PPI* proton pump inhibitor; *SD* standard deviation

**Online Resource Table 5. Risk factor profile for gastrointestinal bleeding in high- and low-risk groups based on gastroprotective agent utilization (before propensity score matching)**

| **Characteristics** | | **High risk group (N=16,450)** | | | **Low risk group (N=46,089)** | | |
| --- | --- | --- | --- | --- | --- | --- | --- |
|  |  | **GPA Non-user (N=3,962)** | **PPI User (N=12,488)** | **p-value** | **GPA Non-user (N=16,109)** | **PPI User (N=29,980)** | **p-value** |
|  |  | **N(%)** | **N(%)** |  | **N(%)** | **N(%)** |  |
| **Major risk variables** | |  |  |  |  |  |  |
|  | Recent major surgery or trauma within 30 days | 1,313 (33.1) | 4,060 (32.5) | 0.462 | 0 (0.0) | 0 (0.0) | - |
|  | Estimated GFR < 30 mL/min | 1,048 (26.5) | 2,525 (20.2) | <.001 | 0 (0.0) | 0 (0.0) | - |
|  | Active malignancy (excluding non-melanoma skin cancer) within 12 months | 921 (23.3) | 2,272 (18.2) | <.001 | 0 (0.0) | 0 (0.0) | - |
|  | Spontaneous bleed requiring hospitalization or transfusion within six months or recurrent bleed | 891 (22.5) | 3,869 (31.0) | <.001 | 0 (0.0) | 0 (0.0) | - |
|  | Moderate or severe ischemic stroke within six months | 294 (7.4) | 1,259 (10.1) | <.001 | 0 (0.0) | 0 (0.0) | - |
|  | Previous spontaneous ICH | 52 (1.3) | 184 (1.5) | 0.458 | 0 (0.0) | 0 (0.0) | - |
|  | Previous traumatic ICH within the past 12 months | 1 (0.0) | 10 (0.1) | 0.479* | 0 (0.0) | 0 (0.0) | - |
|  | Cirrhosis with portal hypertension | 2 (0.1) | 16 (0.1) | 0.274* | 0 (0.0) | 0 (0.0) | - |
| **Minor risk variables** | |  |  |  |  |  |  |
|  | Age≥75 | 1,352 (34.1) | 5,558 (44.5) | <.001 | 2,229 (13.8) | 5,439 (18.1) | <.001 |
|  | Chronic use of NSAIDs or steroids | 605 (15.3) | 3,783 (30.3) | <.001 | 569 (3.5) | 2,820 (9.4) | <.001 |
|  | Spontaneous bleed requiring hospitalization or transfusion within 12 months not meeting major feature | 98 (2.5) | 372 (3.0) | 0.096 | 64 (0.4) | 114 (0.4) | 0.779 |

* Fisher's exact test

*GFR* glomerular filtration rate; *GPA* gastroprotective agents; *ICH* intracerebral hemorrhage; *NSAID* non-steroidal anti-inflammatory drug; *PPI* proton pump inhibitor

**Online Resource Table 6. Risk factor profile for gastrointestinal bleeding in high- and low-risk groups based on gastroprotective agent utilization (after propensity score matching)**

|  | | **High risk group (N=7,924)** | | | **Low risk group (N=32,218)** | | |
| --- | --- | --- | --- | --- | --- | --- | --- |
|  |  | **GPA Non-user (N=3,962)** | **PPI User (N=3,962)** | **SMD** | **GPA Non-user (N=16,109)** | **PPI User (N=16,109)** | **SMD** |
|  |  | **N(%)** | **N(%)** |  | **N(%)** | **N(%)** |  |
| **Major risk variables** | |  |  |  |  |  |  |
|  | Recent major surgery or trauma within 30 days | 1,313 (33.1) | 1,470 (37.1) | 0.084 | 0 (0.0) | 0 (0.0) | **-** |
|  | Estimated GFR < 30 mL/min | 1,048 (26.5) | 912 (23.0) | 0.081 | 0 (0.0) | 0 (0.0) | **-** |
|  | Active malignancy (excluding non-melanoma skin cancer) within 12 months | 921 (23.3) | 843 (21.3) | 0.048 | 0 (0.0) | 0 (0.0) | **-** |
|  | Spontaneous bleed requiring hospitalization or transfusion within six months or recurrent bleed | 891 (22.5) | 891 (22.5) | 0.000 | 0 (0.0) | 0 (0.0) | **-** |
|  | Moderate or severe ischemic stroke within six months | 294 (7.4) | 352 (8.9) | 0.055 | 0 (0.0) | 0 (0.0) | **-** |
|  | Previous spontaneous ICH | 52 (1.3) | 63 (1.6) | 0.025 | 0 (0.0) | 0 (0.0) | **-** |
|  | Previous traumatic ICH within the past 12 months | 1 (0.0) | 4 (0.1) | 0.045 | 0 (0.0) | 0 (0.0) | **-** |
|  | Cirrhosis with portal hypertension | 2 (0.1) | 7 (0.2) | 0.026 | 0 (0.0) | 0 (0.0) | **-** |
| **Minor risk variables** | |  |  |  |  |  |  |
|  | Age≥75 | 1,352 (34.1) | 1,440 (36.4) | 0.048 | 2,229 (13.8) | 2,742 (17.0) | 0.089 |
|  | Chronic use of NSAIDs or steroids | 605 (15.3) | 605 (15.3) | 0.000 | 569 (3.5) | 569 (3.5) | 0.000 |
|  | Spontaneous bleed requiring hospitalization or transfusion within 12 months not meeting major feature | 98 (2.5) | 116 (2.9) | 0.025 | 64 (0.4) | 63 (0.4) | 0.000 |

SMD values are presented as absolute values (|SMD|).

*GFR* glomerular filtration rate; *GPA* gastroprotective agents; *ICH* intracerebral hemorrhage; *NSAID* non-steroidal anti-inflammatory drug; *PPI* proton pump inhibitor; *SMD* standardized mean difference

**Online Resource Table 7. Multivariate analysis of dual antiplatelet therapy-induced gastrointestinal bleeding by risk group**

| **Characteristics** | | **High risk group** | | **Low risk group** | |
| --- | --- | --- | --- | --- | --- |
|  |  | **aHR^a^** | **95% CI** | **aHR^b^** | **95% CI** |
| **Age≥75 years** | | 1.72 | (1.27, 2.33) | 1.20 | (0.94, 1.52) |
| **Sex, male** | | 0.81 | (0.58, 1.14) | 0.77 | (0.61, 0.98) |
| **DAPT combination** | |  |  |  |  |
|  | Aspirin/clopidogrel | Reference | Reference | Reference | Reference |
|  | Aspirin/prasugrel | 1.07 | (0.50, 2.29) | 1.40 | (1.03, 1.90) |
|  | Aspirin/ticagrelor | 1.25 | (0.91, 1.72) | 1.19 | (0.99, 1.43) |
| **Gastroprotective agent types** | |  |  |  |  |
|  | GPA non-user | Reference | Reference | Reference | Reference |
|  | PPI user | 0.74 | (0.56, 0.98) | 0.90 | (0.76, 1.07) |
| **Comorbidities** | |  |  |  |  |
|  | Cirrhosis with portal hypertension | 6.44 | (0.85, 48.88) | - | (-, -) |
|  | Chronic kidney disease | 1.61 | (1.18, 2.22) | 0.69 | (0.17, 2.77) |
|  | Diabetes mellitus | 1.04 | (0.78, 1.38) | 1.21 | (1.01, 1.45) |
|  | Dyspepsia | 1.08 | (0.78, 1.49) | 1.10 | (0.90, 1.35) |
|  | Heart failure | 1.05 | (0.67, 1.64) | 0.98 | (0.65, 1.46) |
|  | Hemorrhagic stroke | 0.86 | (0.21, 3.50) | 1.66 | (0.23, 11.81) |
| **Co-medications** | |  |  |  |  |
|  | Corticosteroid | 1.28 | (0.87, 1.89) | 1.12 | (0.84, 1.49) |
|  | Selective serotonin reuptake inhibitor | 1.46 | (0.79, 2.70) | 1.51 | (0.89, 2.56) |

^a^ Adjusted by age≥75 years, sex, dual antiplatelet therapy combination, cirrhosis with portal hypertension, chronic kidney disease, diabetes mellitus, dyspepsia, heart failure, hemorrhagic stroke, corticosteroid, and selective serotonin reuptake inhibitor

^b^ Adjusted by age≥75 years, sex, dual antiplatelet therapy combination, chronic kidney disease, diabetes mellitus, dyspepsia, heart failure, hemorrhagic stroke, corticosteroid, and selective serotonin reuptake inhibitor

*aHR* adjusted hazard ratio; *CI* confidence interval; *DAPT* dual antiplatelet therapy; *PPI* proton pump inhibitor

**Online Resource Table 8. Assigned and actual gastroprotective agent cohort distribution over time in high and low-risk groups**

| **Cohort** | | **Metric** | **Baseline (0M)** | **6 Months** | **1 Year** | **2 Years** | **3 Years** |
| --- | --- | --- | --- | --- | --- | --- | --- |
| **High risk** | |  |  |  |  |  |  |
|  | GPA non-user | Assigned | 3,962 | 3,124 | 2,680 | 697 | 148 |
|  |  | Actual | 3,962 | 3,121 | 2,673 | 697 | 148 |
|  | PPI user | Assigned | 3,962 | 3,174 | 2,720 | 757 | 131 |
|  |  | Actual | 3,962 | 3,175 | 2,723 | 757 | 131 |
|  | H2RA user | Assigned | 0 | 0 | 0 | 0 | 0 |
|  |  | Actual | 0 | 2 | 4 | 0 | 0 |
| **Low risk** | |  |  |  |  |  |  |
|  | GPA non-user | Assigned | 16,109 | 13,832 | 12,288 | 3,198 | 772 |
|  |  | Actual | 16,109 | 13,825 | 12,277 | 3,190 | 767 |
|  | PPI user | Assigned | 16,109 | 14,114 | 12,514 | 3,474 | 761 |
|  |  | Actual | 16,109 | 14,117 | 12,520 | 3,477 | 763 |
|  | H2RA user | Assigned | 0 | 0 | 0 | 0 | 0 |
|  |  | Actual | 0 | 4 | 5 | 5 | 3 |

*GPA* gastroprotective agent; *H2RA* histamine-2 receptor antagonist; *PPI* proton pump inhibitor

**Online Resource Table 9. Sensitivity analysis using interaction terms between proton pump inhibitor use and bleeding risk status in the pooled cohort**

| **Outcome** | **Risk group** | **Adjusted HR (95% CI)^a^** | **p-value** | **Interaction p-value** |
| --- | --- | --- | --- | --- |
|  |  |  |  |  |
| **GI bleeding** | High risk | 0.75 (0.57, 0.99) | 0.042 | 0.288 |
|  | Low risk | 0.89 (0.76, 1.06) | 0.191 |  |
| **MACE** | High risk | 1.06 (0.92, 1.23) | 0.401 | 0.794 |
|  | Low risk | 1.04 (0.96, 1.12) | 0.303 |  |

Hazard ratios and p-values were derived from pooled multivariable Cox regression models including an interaction term between PPI use and bleeding risk status. Interaction terms assess the heterogeneity of PPI effects across risk groups.

^a^ Adjusted by age ≥ 75 years, sex, dual antiplatelet therapy combination, chronic kidney disease, diabetes mellitus, dyspepsia, heart failure, hemorrhagic stroke, corticosteroid, and selective serotonin reuptake inhibitor

*CI* confidence interval; *GI* gastrointestinal; *HR* hazard ratio; *MACE* major adverse cardiovascular event; *PPI* proton pump inhibitor
